# Genome-wide association study for intramuscular fat content in Chinese Lulai black pigs

**DOI:** 10.5713/ajas.18.0483

**Published:** 2018-10-26

**Authors:** Yanping Wang, Chao Ning, Cheng Wang, Jianfeng Guo, Jiying Wang, Ying Wu

**Affiliations:** 1 Shandong Provincial Key Laboratory of Animal Disease Control and Breeding, Institute of Animal Science and Veterinary Medicine, Shandong Academy of Agricultural Sciences, Jinan 250100, China; 2 Key Laboratory of Animal Genetics, Breeding and Reproduction, Ministry of Agriculture, College of Animal Science and Technology, China Agricultural University, Beijing 100193, China

**Keywords:** Genome-wide Association Study, Intramuscular Fat Content, Single Nucleotide Polymorphisms, Pigs

## Abstract

**Objective:**

Intramuscular fat (IMF) content plays an important role in meat quality. Identification of single nucleotide polymorphisms (SNPs) and genes related to pig IMF, especially using pig populations with high IMF content variation, can help to establish novel molecular breeding tools for optimizing IMF in pork and unveil the mechanisms that underlie fat metabolism.

**Methods:**

We collected muscle samples of 453 Chinese Lulai black pigs, measured IMF content by Soxhlet petroleum-ether extraction method, and genotyped genome-wide SNPs using GeneSeek Genomic Profiler Porcine HD BeadChip. Then a genome-wide association study was performed using a linear mixed model implemented in the GEMMA software.

**Results:**

A total of 43 SNPs were identified to be significantly associated with IMF content by the cutoff p<0.001. Among these significant SNPs, the greatest number of SNPs (n = 19) were detected on Chr.9, and two linkage disequilibrium blocks were formed among them. Additionally, 17 significant SNPs are mapped to previously reported quantitative trait loci (QTLs) of IMF and confirmed previous QTLs studies. Forty-two annotated genes centering these significant SNPs were obtained from Ensembl database. Overrepresentation test of pathways and gene ontology (GO) terms revealed some enriched reactome pathways and GO terms, which mainly involved regulation of basic material transport, energy metabolic process and signaling pathway.

**Conclusion:**

These findings improve our understanding of the genetic architecture of IMF content in pork and facilitate the follow-up study of fine-mapping genes that influence fat deposition in muscle.

## INTRODUCTION

Pork is an important dietary source for humans, accounting for about 40% of the world’s meat consumption. Its quality has a great influence on consumer preferences. It is generally accepted that a high level of marbling or intramuscular fat (IMF) content has a positive influence on the eating quality of pork [1]. Over the last decades, pig selection has mainly focused on growth traits and lean meat rate. Due to the reverse correlation between lean meat rate and IMF content, current commercial pigs deposit less fat in backfat as well as muscle at market weights than native breeds. Therefore, the pork industry is greatly interested in augmenting the IMF content to satisfy the eating experience of the consumer.

IMF content displays substantial genetic variations between breeds and even lines within breed [2,3]. Although large differences exist, improvement by normal breeding programs is challenging due to the difficulty of their phenotypic measurement. The measurement of the trait is not only time-consuming and costly, but samples are collected only after slaughtering the pigs. Genetic selection is generally believed to be a promising approach to improve the trait. Thus, it is worthwhile to study the genetic mechanisms underlying IMF content, which would allow us to establish novel molecular breeding tools for its optimization in pork. Furthermore, since the pig is an excellent animal model to study various human diseases [4], dissection of the genetic architecture of fat deposition in porcine muscle can also provide information for unveiling the mechanisms that underlie fat metabolism in humans.

Over the past two decades, using microsatellite markers, many quantitative trait loci (QTLs) that contribute to the IMF content [5,6] have been detected in pigs. However, due to the low density of microsatellite markers, these QTLs represent large chromosomal regions, and further fine-mapping studies are necessary to find the causal mutation responsible for these effects. With the development of high-throughput single nucleotide polymorphism (SNP) genotyping methods, pig-specific high-density genotyping chips have become available. Genome-wide association studies (GWAS), using the chips, makes it possible to detect the genetic variants underlying the traits of interest. Recently, several GWAS have made substantial progresses in identifying genetic factors associated with or underlying a variety of traits, including IMF content [7–10]. However, compared with other traits, studies dissecting variations for IMF content are still rare. Therefore, research should further investigate the genetic mechanism of IMF content, especially using pig populations with high IMF content variation.

Lulai black pigs are a newly developed breed which originated from a cross between Chinese Laiwu pigs (a typical Chinese indigenous breed) and Yorkshire pigs, and then artificially selected for more than eight generations. This breed, similar as Laiwu pigs, is known for its high IMF content [11,12]. Here, to unveil the genetic architecture of IMF content of pork, we measured IMF content of Lulai black pigs, and conducted GWAS for the trait.

## MATERIALS AND METHODS

### Ethics statement

The whole study protocols for animal rearing and slaughter were reviewed and approved by the guideline (IACC20060101, 1 January 2006) of the Institutional Animal Care and Use Committee of Institute of Animal Science and Veterinary Medicine, Shandong Academy of Agricultural Sciences.

### Animal resource

Four hundred and eighty Lulai black pigs were purchased from nucleus farms of the breed in Laiwu, Shandong province. These pigs originated from 12 sires and 95 dams, representing offspring of all boars and most sows in the farm. Boars were castrated before day 60. All pigs had *ad libitum* access to a corn-soybean based diet containing 13.3% crude protein, 12.43 MJ/kg digestible energy and 0.80% lysine under standard management conditions. Out of the 480 pigs, 453 pigs, including 289 males and 164 females, grew well and were slaughtered when their weights were in the range of 70 to 100 kg. About 200 g longissimus dorsi muscle was collected from the last rib of each pig and stored at −20°C for further measurement.

### Measurement of intramuscular fat content

After removing adipose and connective tissue, the longissimus dorsi muscle was ground and analyzed for moisture by routine oven-drying method. Then, samples that had already been analyzed for moisture were ground into powder, which were further used for measuring IMF content. IMF content was determined using the Soxhlet petroleum-ether extraction method and expressed as the weight percentage of wet muscle tissue.

### Single nucleotide polymorphism array genotyping and quality control

A standard phenol/chloroform method was used to extract genomic DNA from muscle samples for all the pigs. The quality and concentration of genomic DNA fulfilled the requirements for the Illumina SNP genotyping platform. Samples were genotyped with GeneSeek Genomic Profiler Porcine HD BeadChip (Neogen Corporation, Lansing, MI, USA) according to the manufacturer’s protocol and genotypes were called using GenomeStudio (version 2011.1; Illumina Inc., San Diego, CA, USA).

Quality controls were implemented by Plink v1.07 [13] according to the following filtering. Firstly, SNPs with GC score below 0.2 were considered failed genotypes, and Fimpute [14] was used to impute the failed loci. Then, SNPs were excluded from the data set if i) SNPs without genome location based on the pig genome assembly Sus scrofa Build 10.2 or located on Y chromosome, ii) its minor allele frequency was <5%, or iii) it departed severely from Hardy–Weinberg equilibrium with a p-value lower than 10^6^.

### Statistical analysis

The association analysis was performed using the software GEMMA [15]. SNPs were individually tested for association with IMF content using the following linear mixed model:

Y=Xf+Zp+kg+e

where **Y** is the vector of phenotypic values of IMF content; X is the incidence matrices of fixed effects including population mean, sex and weight, and **f** is the vector for these fixed effects; **Z** is the identity matrix, and **p** is the vector for polygenic effects with distribution of $N(0,G\sigma_{a}^{2})$, where G is the genomic relationship matrix that was constructed based on genome-wide SNP markers and $\sigma_{a}^{2}$ the additive genetic variance; **k** is the vector of 0, 1, 2 values, where 0 and 2 correspond to the two homozygous genotypes and 1 represents the heterozygous genotype at the tested SNP, and g is the additive genetic effect of the tested SNP; and **e** is the vector of residuals.

Firstly, permutation was adopted to adjust for multiple testing for the number of SNPs tested through constructing an empirical distribution of the test statistic under the null hypothesis. Specifically, the phenotypic observations of each trait were randomly shuffled 10,000 times, and the empirical critical values for chromosome-wise and genome-wise significance were determined by the 95th percentile of the highest test statistic over the 10,000 permutation replicates. Then, to detect significantly associated SNPs in a study with low power, a liberal cutoff (raw p-value and p<0.001) was used as the cutoff for statistical significance of SNPs. The Q-Q plot and linkage disequilibrium (LD) analysis of the significant SNPs were constructed using the R software and Haploview [16], respectively.

### Characterization of candidate genes

To identify plausible candidate genes, we retrieved annotated genes within a 50 kb region centering each significant SNP from Ensembl Genes 89 Database using BioMart (http://asia.ensembl.org/biomart/martview/). Functional analyses, including gene ontology (GO) and reactome pathway enrichment, were performed using PANTHER 13.1 (Feb. 2018) (http://www.pantherdb.org/) to reveal the potential biological function of annotated genes. Furthermore, QTLs of IMF were downloaded from the AnimalQTL database (www.animalgenome.org, Release 34, Dec 21, 2017), and compared with those significant SNPs based on the putative location of these QTLs.

## RESULTS

### Summary of phenotypic and single nucleotide polymorphism data

**Figure 1:**
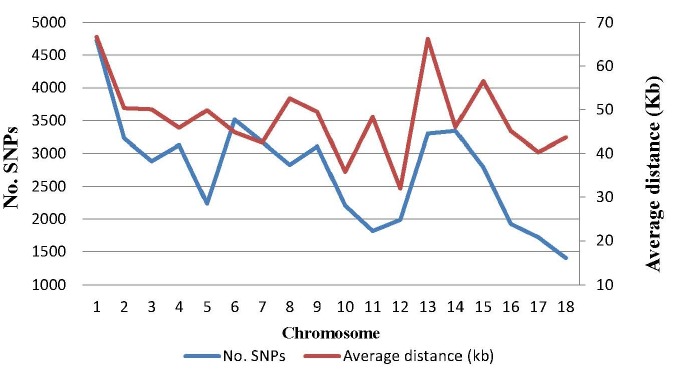
Distribution of SNPs after quality control and the average distances between adjacent SNPs on each chromosome. SNPs, single nucleotide polymorphisms.

The mean and variation coefficient of the phenotypic observations of the IMF content were 5.18% and 65.07%, respectively. The IMF content increased significantly with the weight increment (p<0.05), with the average of 4.80% for weight range 70 to 80 kg, 4.94% for 80 to 90 kg and 5.93% for 90 to 100 kg, respectively. On the other hand, out of the 68,516 SNPs genotyped, a final set of 49,383 SNPs passed the filters and was retained in the data set for further statistical analyses. The distribution of SNP markers and marker density on autosomes are shown in Figure 1. The number of SNPs on every chromosome varies from 1,403 on Chr.18 to 4,728 on Chr.1, and the adjacent distance ranges from 32.00 Kb on Chr.12 to 66.65 Kb on Chr.1. Compared with the common used Porcine SNP60 BeadChip in previous study [17,18], this porcine BeadChip has more usable, and evenly distributed SNPs.

### Significantly associated single nucleotide polymorphisms identified in genome-wide association study

**Figure 2:**
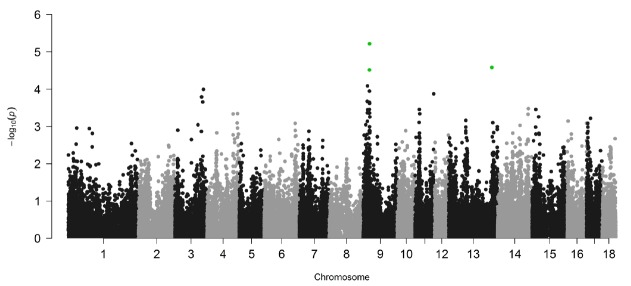
Genome-wide plots of −log_10_(*P*) for the association of SNPs with intramuscular fat content. Green dots denote the significant SNPs at chromosome-level after permutation test. SNPs, single nucleotide polymorphisms.

Linear mixed models implemented in GEMMA [15] were used in the study to perform the association analysis of IMF content and SNPs. To adjust the effect of inbreeding, a genetic relationship matrix was constructed and used during the analysis. The profile plot of the p values (in terms of −log_10_(*p*)) for IMF content is shown in Figure 2. Q-Q plot of the tested SNPs is provided in Supplementary Figure S1, and the SNPs tested show no evidence of overall systematic bias.

**Figure 3:**
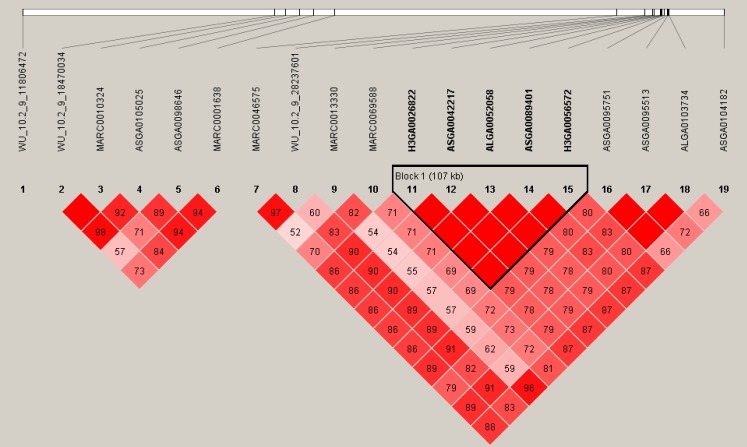
The LD pattern for the significant SNPs associated with intramuscular fat content on chr. 9. Values in boxes are LD (r^2^) between SNP pairs and the boxes are colored according to the standard Haploview color scheme. LD, linkage disequilibrium; SNPs, single nucleotide polymorphisms.

By the cutoff p<0.001, a total of 43 SNPs from 18 autosomes were identified to be significantly associated with IMF content, and the details of the significant SNPs are given in Table 1. Among these significant SNPs, the greatest number (n = 19) was detected on Chr.9. The LD block analysis (Figure 3) by Haploview 4.2 [16] indicated that there are two LD blocks formed among these significant SNPs on Chr.9. Especially in the larger LD, five SNPs are in the complete linkage.

### Functional characterization of significant single nucleotide polymorphisms

Among the 43 significant SNPs identified in the study, there are 15 significant SNPs located in the protein coding sequences, while the others are mapped in the non-coding sequences. It is consistent with the previous studies that the clear majority of significantly associated SNPs reside outside the protein-coding regions, underscoring the important function of non-coding sequences in the genome [19].

Association studies essentially identify a genomic location related to phenotypic traits but provide little functional significance, especially those significant SNPs occurring in non-coding sequences. To identify possible candidate genes related with IMF content, a total of 42 annotated genes (Supplementary Table S1) centering the significant SNPs detected were obtained from Ensembl database. Overrepresentation test of reactome pathways and GO terms using PANTHER revealed some enriched reactome pathways and GO terms, which are provided in Supplementary Table S2 and S3. Most of the terms and pathways were involved regulation of basic material transport, energy metabolic process and signaling pathway.

To test whether the significant SNPs were mapping into QTLs of IMF detected in previous studies, we downloaded QTLs of IMF from the pig QTL database and compared by their physical positions. Consequently, these are 17 significant SNPs (Supplementary Table S4) mapped to 6 QTLs of IMF.

## DISCUSSION

In the present study, we measured IMF content of 453 Lulai black pigs. Consistent with previous reports [11,20], our results suggested that Lulai black pigs had high capability in depositing IMF, with the average of 5.18%. The IMF content increased significantly with the weight increment, which was also reported in the previous studies [20,21], indicating IMF is deposited at a greater rate when pigs get older. Furthermore, the population variation of IMF content was rather large, with variation coefficient of 65.07%, suggesting genotypes of SNPs affecting IMF content are still segregating in the population.

One of the major challenges in GWAS is multiple hypothesis testing. Permutation test is the gold standard in multiple testing corrections [22]. In our study, permutation was firstly used to adjust multiple testing. However, no significant SNPs were identified to be associated with IMF content at the genome-wide significance level, and only three SNPs (green dots in Figure 1) were found at chromosome-wide significance. The specific samples used in this study may be one possible reason leading to the few significant SNPs detected. As mentioned above, Lulai black pigs originated from a cross between typical Chinese Laiwu pigs and Yorkshire pigs. So, they have a complicated genetic background, and strict adjustments were implemented to correct potential effects of population structure in the analyses using GEMMA, which may decrease the significant SNPs identified. The other reason may be the smaller sample size used in our study. In our study, the sample size used (453 Lulai pigs) is small, which reduces the power to detect SNPs related to IMF contents. Consequently, to detect significantly associated SNPs in the study, a liberal cutoff (raw p value and p<0.001) was taken instead of generally used multiple testing correction method. This might cause some significant SNPs to be false positives. But, as proved by mapping with previously reported QTLs and functional analyses of annotated genes centering significant SNP, the SNPs detected by liberal p value in this study can be suggested as SNPs affecting IMF content.

Furthermore, part (n = 17) of the significant SNPs identified are mapped to previously reported QTLs of IMF. However, compared with the previous GWAS, our results do not replicate the significant SNPs identified by them. The different SNP chips and genome assembly used may be possible reasons. However, even using the same SNP chips, a lack of reproducibility of genetic associations has been frequently observed in GWAS [23]. For instance, using Illumina PorcineSNP60 Beadchip, Luo et al [8] identified 40 significant SNPs for IMF in a porcine Large White×Minzhu intercross population, while Ma et al [24] detected 7 and 25 significant SNPs for IMF in the Duroc×Erhualian F2 animals and Chinese Sutai pigs. Of note, no common SNPs for IMF were found in the two populations, Duroc×Erhualian F2 animals identified in Large White×Minzhu intercross population was confirmed by Ma et al [24]. One reason for the lower reproducibility is that GWAS can generate some false positive and false negative associations, although sophisticated statistical tests have been proposed to reduce false positives. Another possible reason is that IMF content is complex trait, for which gene-gene and gene-environment interactions possibly have different genetic effects across population. Therefore, further confirmation of these associations in larger independent populations would be warranted to test if the significant SNPs identified in this study are population-specific before their incorporation into breeding programs.

## Supplementary Data



## Figures and Tables

**Table 1:** Detailed information of SNPs significantly associated with IMF content in Lulai black pigs

SNP name	Chr.	Position	MAF	p-value	Nearest genes	Distance (bp)
ALGA0020253	3	102968480	0.062	9.02E-04	ENSSSCG00000028599	Within
ASGA0016143	3	119316058	0.053	1.61E-04	ENSSSCG00000008563	21,437
ALGA0021205	3	124747830	0.063	2.21E-04	ENSSSCG00000008595	480,221
H3GA0054493	3	127607014	0.083	1.01E-04	RDH14	64,442
WU_10.2_4_116001597	4	116001597	0.053	4.59E-04	CSDE1	Within
H3GA0014557	4	134029125	0.053	8.08E-04	RWDD3	34,414
ALGA0029232	4	135638341	0.063	4.50E-04	ENSSSCG00000006897	Within
WU_10.2_6_139169213	6	139169213	0.205	8.25E-04	ENSSSCG00000023243	404,669
WU_10.2_9_11806472	9	11806472	0.132	8.96E-04	EMSY	Within
WU_10.2_9_18470034	9	18470034	0.185	2.12E-04	ENSSSCG00000024491	20,269
MARC0010324	9	18740429	0.148	4.31E-04	FAM181B	43,106
ASGA0105025	9	19113660	0.149	7.28E-04	ENSSSCG00000014900	4,203
ASGA0098646	9	19497181	0.266	8.14E-05	DLG2	Within
MARC0001638	9	20050249	0.300	3.57E-04	ENSSSCG00000014904	572,013
MARC0046575	9	27497398	0.342	4.43E-04	FAT3	647,311
WU_10.2_9_28237601	9	28237601	0.323	3.49E-04	FAT3	Within
MARC0013330	9	28446168	0.395	3.03E-05	FAT3	Within
MARC0069588	9	28476184	0.409	1.12E-04	FAT3	Within
H3GA0026822	9	28642010	0.304	2.25E-04	FAT3	Within
ASGA0042217	9	28671488	0.304	2.25E-04	FAT3	Within
ALGA0052058	9	28682617	0.304	2.25E-04	FAT3	Within
ASGA0089401	9	28741033	0.294	4.35E-04	ENSSSCG00000022342	13,207
H3GA0056572	9	28749221	0.294	4.35E-04	ENSSSCG00000022342	5,019
ASGA0095751	9	28818688	0.285	2.41E-04	ENSSSCG00000014939	19,229
ASGA0095513	9	28845096	0.321	6.09E-06	ENSSSCG00000014939	45,637
ALGA0103734	9	28872956	0.285	2.41E-04	ENSSSCG00000026565	36,805
ASGA0104182	9	30319497	0.178	5.54E-04	MRE11	9,526
ALGA0061169	11	18669144	0.195	7.80E-04	CAB39L	Within
WU_10.2_11_19501392	11	19501392	0.185	3.48E-04	ENSSSCG00000018211	21,006
WU_10.2_11_20807311	11	20807311	0.163	4.56E-04	HTR2A	88,971
WU_10.2_11_83286917	11	83286917	0.063	1.33E-04	ENSSSCG00000009543	141,789
ASGA0058053	13	76143725	0.341	6.90E-04	ENSSSCG00000011591	3,040
ALGA0073197	13	192200740	0.071	2.61E-05	ENSSSCG00000012012	84,101
ALGA0122724	13	196766412	0.299	7.93E-04	ENSSSCG00000012016	4,105,434
ALGA0079891	14	99642277	0.411	9.33E-04	LOC100512136	Within
WU_10.2_14_135918985	14	135918985	0.196	4.72E-04	ABLIM1	Within
H3GA0042485	14	137063119	0.255	3.32E-04	ENSSSCG00000021667	386,100
WU_10.2_15_16929105	15	16929105	0.127	3.49E-04	ENSSSCG00000015724	1,247,209
ALGA0117841	15	29674589	0.500	5.53E-04	GYPC	79,387
WU_10.2_16_3543276	16	3543276	0.432	7.18E-04	ENSSSCG00000029792	292,955
ASGA0074877	16	86742597	0.398	8.18E-04	ENSSSCG00000017123	3,804
WU_10.2_17_5301556	17	5301556	0.205	8.24E-04	ZDHHC2	Within
WU_10.2_17_17145778	17	17145778	0.459	6.08E-04	ENSSSCG00000022670	93,169

SNP, single nucleotide polymorphism; IMF, intramuscular fat; MAF, minor allele frequency.
