# Supplementary material for: Genome-wide association study for intramuscular fat content in Chinese Lulai black pigs
Source: Asian-Australas J Anim Sci. 2018 Oct 26;32(5):607–13. doi: 10.5713/ajas.18.0483 (PMC6502724; doi:10.5713/ajas.18.0483)
Supplement: Supplementary file 1 [file ajas-18-0483-suppl.pdf]

S1 Figure

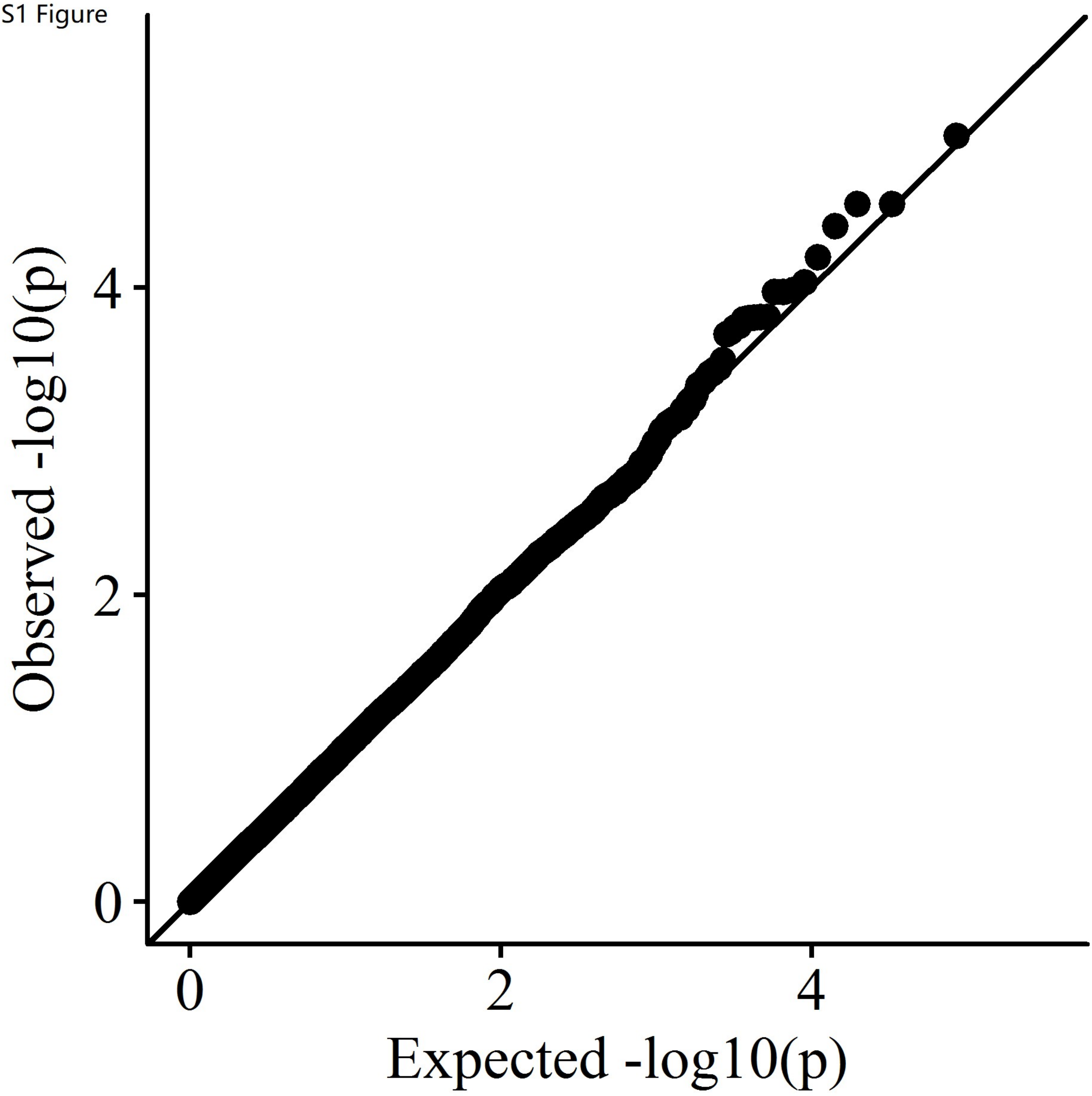

Supplementary Figure S1. Quantile-quantile (Q-Q) plot of the genome-wide association result for IMF content.

Supplementary Table S1. Annotated genes centering the significant SNPs detected

Gene stable ID	Chr.	Gene start (bp)	Gene end (bp)	Gene name	Gene type
ENSSSCG00000028599	3	102939970	103176416		protein_coding
ENSSSCG00000008561	3	119265407	119272400	CENPA	protein_coding
ENSSSCG00000008562	3	119279961	119296228	SLC35F6	protein_coding
ENSSSCG00000008563	3	119337495	119338417		pseudogene
ENSSSCG00000006751	4	115968345	115974919	SIKE1	protein_coding
ENSSSCG00000006752	4	115990249	116034509	CSDE1	protein_coding
ENSSSCG00000006753	4	116034666	116042522	NRAS	protein_coding
ENSSSCG00000006883	4	133994711	133998553	RWDD3	protein_coding
ENSSSCG00000006897	4	135595935	135692379		protein_coding
ENSSSCG00000006898	4	135640163	135752386	MTF2	protein_coding
ENSSSCG00000014868	9	11731924	11824724	EMSY	protein_coding
ENSSSCG00000024491	9	18490303	18490490	U2	snRNA
ENSSSCG00000014897	9	18783535	18785418	FAM181B	protein_coding
ENSSSCG00000014900	9	19117863	19130117		protein_coding
ENSSSCG00000014904	9	19478236	19503322		protein_coding
ENSSSCG00000014935	9	28144709	28725103	FAT3	protein_coding
ENSSSCG00000022342	9	28754240	28756842		protein_coding
ENSSSCG00000014938	9	28768923	28769272		pseudogene
ENSSSCG00000014939	9	28799459	28811592		protein_coding
ENSSSCG00000026565	9	28909761	28910107		pseudogene
ENSSSCG00000024577	9	28922157	28924779		protein_coding
ENSSSCG00000014952	9	30213182	30277222	IZUMO1R	protein_coding
ENSSSCG00000022490	9	30303203	30317325	GPR83	protein_coding
ENSSSCG00000014955	9	30329023	30388681	MRE11	protein_coding
ENSSSCG00000020861	9	30356729	30356835	U6	snRNA
ENSSSCG00000021060	11	18464851	18512551	RCBTB1	protein_coding
ENSSSCG00000009397	11	18628807	18716116	CAB39L	protein_coding
ENSSSCG00000029933	11	19056603	19200872		protein_coding
ENSSSCG00000009399	11	19465520	19487745	CYSLTR2	protein_coding
ENSSSCG00000018211	11	19480386	19480492	U6	snRNA
ENSSSCG00000011589	13	76112280	76129343		protein_coding
ENSSSCG00000011590	13	76135867	76140946	RHO	protein_coding
ENSSSCG00000011591	13	76146765	76175465		protein_coding
ENSSSCG00000011592	13	76179149	76229571	PLXND1	protein_coding
ENSSSCG00000010411	14	99566737	99612051		protein_coding
ENSSSCG00000025787	14	99624054	99643343		protein_coding
ENSSSCG00000010651	14	135884829	136136366	ABLIM1	protein_coding
ENSSSCG00000030984	15	29595202	29625473	GYPC	protein_coding
ENSSSCG00000017123	16	86738793	86739005		protein_coding
ENSSSCG00000017124	16	86762462	86840982		protein_coding
ENSSSCG00000006982	17	5281876	5322408	ZDHHC2	protein_coding
ENSSSCG00000006983	17	5328135	5343368	CNOT7	protein_coding

Supplementary Table S2. Significantly enriched reactome pathways of annotated genes

Reactome pathways	Reference	Observed	Fold Enrichment	P value
Activation of the phototransduction cascade (R-SSC-2485179)	3	1	> 100	6.46E-03
Class A/1 (Rhodopsin-like receptors) (R-SSC-373076)	262	3	7.06	8.95E-03
SUMO is transferred from E1 to E2 (UBE2I, UBC9) (R-SSC-3065678)	5	1	> 100	9.68E-03
Leukotriene receptors (R-SSC-391906)	6	1	> 100	1.13E-02
Opsins (R-SSC-419771)	6	1	> 100	1.13E-02
Sensing of DNA Double Strand Breaks (R-SSC-5693548)	8	1	77.05	1.45E-02
Processing and activation of SUMO (R-SSC-3215018)	10	1	61.64	1.77E-02
GPCR ligand binding (R-SSC-500792)	340	3	5.44	1.79E-02
Inactivation, recovery and regulation of the phototransduction cascade (R-SSC-2514859)	12	1	51.37	2.09E-02
The phototransduction cascade (R-SSC-2514856)	13	1	47.42	2.24E-02
Other semaphorin interactions (R-SSC-416700)	13	1	47.42	2.24E-02
HDR through MMEJ (alt-NHEJ) (R-SSC-5685939)	13	1	47.42	2.24E-02
Eicosanoid ligand-binding receptors (R-SSC-391903)	14	1	44.03	2.40E-02
TP53 regulates transcription of additional cell cycle genes whose exact role in the p53 pathway remain uncertain (R-SSC-6804115)	18	1	34.25	3.03E-02
VxPx cargo-targeting to cilium (R-SSC-5620916)	19	1	32.44	3.19E-02
Cilium Assembly (R-SSC-5617833)	173	2	7.13	3.26E-02
The canonical retinoid cycle in rods (twilight vision) (R-SSC-2453902)	20	1	30.82	3.35E-02
Organelle biogenesis and maintenance (R-SSC-1852241)	195	2	6.32	4.04E-02
TP53 Regulates Transcription of Cell Cycle Genes (R-SSC-6791312)	27	1	22.83	4.44E-02
Surfactant metabolism (R-SSC-5683826)	28	1	22.01	4.59E-02
PRC2 methylates histones and DNA (R-SSC-212300)	29	1	21.26	4.75E-02
Cytosolic sensors of pathogen-associated DNA (R-SSC-1834949)	29	1	21.26	4.75E-02

Supplementary Table S3. Significantly enriched GO terms of annotated genes

PANTHER GO terms	Reference	Observed	Fold Enrichment	raw P value
GTPase activity (GO:0003924)	400	5	7.71	4.64E-04
translation elongation factor activity (GO:0003746)	24	2	51.37	8.09E-04
chromatin binding (GO:0003682)	203	3	9.11	4.47E-03
translation regulator activity (GO:0045182)	77	2	16.01	7.27E-03
chromatin organization (GO:0006325)	258	3	7.17	8.59E-03
cilium (GO:0005929)	86	2	14.34	8.95E-03
protein localization (GO:0008104)	547	4	4.51	1.17E-02
cellular component organization (GO:0016043)	2005	8	2.46	1.35E-02
cell adhesion (GO:0007155)	357	3	5.18	2.03E-02
biological adhesion (GO:0022610)	357	3	5.18	2.03E-02
cellular component organization or biogenesis (GO:0071840)	2165	8	2.28	2.07E-02
asymmetric protein localization (GO:0008105)	14	1	44.03	2.40E-02
nucleobase-containing compound metabolic process (GO:0006139)	3020	10	2.04	2.43E-02
cell recognition (GO:0008037)	19	1	32.44	3.19E-02
small GTPase regulator activity (GO:0005083)	177	2	6.97	3.40E-02
pyrophosphatase activity (GO:0016462)	760	4	3.24	3.42E-02
exoribonuclease activity (GO:0004532)	22	1	28.02	3.66E-02
localization (GO:0051179)	2059	7	2.1	4.52E-02

Supplementary Table S4. Significant SNPs mapped to previously reported QTLs of IMF

**Significant SNPs**			**QTL information**			
SNP_name	chr.	position	chr.	Start	end	Traits
H3GA0014557	4	134029125	4	123430356	135006664	Intramuscular fat content QTL (3842)
WU_10.2_6_139169213	6	139169213	6	129740986	146365886	Intramuscular fat content QTL (3862)
WU_10.2_9_28237601	9	28237601	9	27564748	98136454	Intramuscular fat content QTL (125483)
MARC0013330	9	28446168	9	27564748	98136454	Intramuscular fat content QTL (125483)
MARC0069588	9	28476184	9	27564748	98136454	Intramuscular fat content QTL (125483)
H3GA0026822	9	28642010	9	27564748	98136454	Intramuscular fat content QTL (125483)
ASGA0042217	9	28671488	9	27564748	98136454	Intramuscular fat content QTL (125483)
			9	27651964	28179642	Intramuscular fat content QTL (9035)
ALGA0052058	9	28682617	9	27564748	98136454	Intramuscular fat content QTL (125483)
			9	27651964	28179642	Intramuscular fat content QTL (9035)
ASGA0089401	9	28741033	9	27564748	98136454	Intramuscular fat content QTL (125483)
H3GA0056572	9	28749221	9	27564748	98136454	Intramuscular fat content QTL (125483)
ASGA0095751	9	28818688	9	27564748	98136454	Intramuscular fat content QTL (125483)
ASGA0095513	9	28845096	9	27564748	98136454	Intramuscular fat content QTL (125483)
ALGA0103734	9	28872956	9	27564748	98136454	Intramuscular fat content QTL (125483)
ASGA0104182	9	30319497	9	27564748	98136454	Intramuscular fat content QTL (125483)
WU_10.2_11_19501392	11	19501392	11	18802133	19542650	Intramuscular fat content QTL (106285)
WU_10.2_17_5301556	17	5301556	17	435637	32082161	Intramuscular fat content QTL (12074)
WU_10.2_17_17145778	17	17145778	17	435637	32082161	Intramuscular fat content QTL (12074)
